# Clozapine Prescribing in a Canadian Outpatient Population

**DOI:** 10.1371/journal.pone.0083539

**Published:** 2013-12-16

**Authors:** Silvia Alessi-Severini, Josee-Anne Le Dorze, David Nguyen, Patricia Honcharik, Michael Eleff

**Affiliations:** 1 Faculty of Pharmacy, University of Manitoba, Winnipeg, Manitoba, Canada; 2 Department of Pharmaceutical Services, Health Sciences Centre, Winnipeg, Manitoba, Canada; 3 Department of Psychiatry, Faculty of Medicine, University of Manitoba, Winnipeg, Manitoba, Canada; Concordia University Wisconsin, United States of America

## Abstract

**Objective:**

Description of demographics of an outpatient population of clozapine users.

**Methods:**

Retrospective chart review study of an urban population diagnosed with schizophrenia. Assessment of therapeutic histories in relation to clinical practice guidelines.

**Results:**

Seventy-seven of the 467 patients were on clozapine therapy. Average patients’ age was 39.4 ± 11.8 years) and 68% were males. The majority of patients (68%) had tried 3 or more antipsychotics before switching to clozapine, 21% had tried two and 11% had tried one. Median length of therapy prior to clozapine initiation was 8.9 years in males and 7.7 years in females.

**Conclusion:**

Until 2010, the use of clozapine was often delayed and more than 2 antipsychotic medications were tried for relatively long periods of time before patients were switched to this effective agent.

## Introduction

Clozapine has been shown to be more effective than both first-generation [1] and second-generation antipsychotic agents (SGAs) [2] in treatment-resistant schizophrenia and in suicide prevention [3]. Patients discharged on clozapine are less likely to be readmitted to hospital [4]; in addition, clozapine is the antipsychotic drug with the lowest rates of discontinuation and switching [5] and is the least likely to require combination with other antipsychotic medications [6].

Current clinical practice guidelines define treatment-resistant schizophrenia as failure to respond to adequate trials with two unrelated antipsychotics [7-10]. However, clinicians are reluctant to prescribe clozapine because of concerns about its safety profile, which includes the well-known risk for agranulocytosis. In fact, clozapine can only be used if regular haematological monitoring can be guaranteed, as per its official monograph [11]. As a result, patients are often subjected to a number of lengthy, ineffective trials before being switched to and stabilized with clozapine [12,13]. 

Our study describes patient characteristics, treatment outcomes and prescription histories in an outpatient population treated with clozapine in the Canadian province of Manitoba. 

## Methods

A retrospective chart-review study was conducted by accessing all available active medical records of outpatients attending psychiatric clinics at the Health Sciences Center Schizophrenia Program (Winnipeg, Manitoba) during the time period May-September 2010. This program provides multidisciplinary service to patients with schizophrenia and their families. Patients 18 years of age and older are accepted into the program following referrals from psychiatrists, community physicians and the Health Sciences Centre Emergency Department. The clinical focus of the program is for complex or treatment-resistant schizophrenia and individuals with co-occurring major mental illness and substance abuse or dependence.

Ethics approval was obtained from the Health Research Ethics Board at the University of Manitoba; due to the non-interventional, retrospective design of the audit, no informed consent was required and in fact it was waived by the HREB. Data were collected in full compliance with the Privacy of Health Information Act legislation in the province of Manitoba. All charts of patients who were on clozapine therapy at the time of data collection were included. Information on demographics, hospitalizations, dosing and adverse events as reported in the charts were recorded in an appropriately designed databases (Microsoft Excel 2007, Microsoft Corp., Redmond, Washington). Patients were assigned a study number and any identifying information was destroyed at the end of the study period. 

## Results

### Demographics

Of the 467 outpatients enrolled in the program, 77 (16%) appeared to be on clozapine therapy at the time of the data collection. Their charts were reviewed and data were collected. Complete information was available for 74 patients of the 77 patients. Patients’ ages ranged from 18 to 73 years (mean +/- SD=39.4 ± 11.8 years) and 68% were males. Age at time of diagnosis was 23.8±8.4 years in males and 25.1±8.1 years in females. Forty per cent of male patients were reported as being smokers as compared to 50% of female patients.

### Therapy

Most patients (60%) had been taking clozapine for more than 5 years, 26% of the patients had been on clozapine therapy between 2 and 5 years; the remainder had been on clozapine therapy less than 2 years (Table 1). The majority of patients (68%) had tried 3 or more antipsychotics before switching to clozapine, 21% had tried two and 11% had tried one. Mean number of antipsychotics tried before clozapine was calculated at 3.3. Median length of therapy prior to clozapine initiation was 8.9 years in males and 7.7 years in females. The most common previously-tried antipsychotic was risperidone (72% of the patient population had reports of prior use). The majority of patients (69%) were on clozapine monotherapy, 31% were taking clozapine along with another antipsychotic medication (most common quetiapine, 11%) and only 3% of patients were managed on three agents (clozapine + risperidone + quetiapine). 

**Table 1:** Demographics of patients on clozapine therapy (N=74).

Sex (N, %)	
Male	52 (68)
Female	22 (32)
Age at time of the study, y, mean ± SD (range 18 - 73 years)	
Male	39.5 ± 11.8
Female	39.2 ± 12.2
Age at time of diagnosis, y, mean ± SD	
Male	23.8 ± 8.4
Female	25.1 ± 8.1
Age at time of clozapine initiation, y, mean ± SD	
Male Female	32.5 ± 11.8 32.8 ± 11.4
Length of clozapine therapy, (N, %)	
< 1year	4 (5.2)
1 to <2 years	5 (6.2)
2 to <5 years	20 (26)
5 years or longer	45 (59.7)
Clozapine dose at re-entry into the outpatient program (N, %)	
<200, mg/day	26 (35)
200 - <300, mg/day	8 (11)
300 - <400, mg/day	22 (30)
400 - <600, mg/day	6 (8)
unknown	12 (16)
Maintenance clozapine dose, (N, %)	
<200, mg/day	4 (5)
200 - <300, mg/day	12 (16)
300 - <400, mg/day	22 (30)
400 - <600, mg/day	23 (31)
600 - <900, mg/day	11 (15)
unknown	2 (3)

A subset of patients (N=22, 28%) who initiated clozapine therapy after 2005 (year of the publication of the most recent Canadian Guidelines for schizophrenia) showed similar results in terms of previous antipsychotic trials: 1(4%), 7(32%) and 14 (64%) participants had tried 1, 2 or 3 or more antipsychotics prior to clozapine initiation. The average length of time from diagnosis to clozapine initiation in this subset of the study population ranged from 3 months to 16 years (mean +/- SD = 4.6 +/- 3.9 years).

Clozapine therapy was normally initiated in the HSC hospital and titrated to optimal dose before patients were discharged to the outpatient program. The mean daily dose of clozapine at re-entry into the outpatient program was 232.8 +/- 106.1 mg. The mean maintenance daily dose was 391.0 +/- 157.7 mg (Table 1).

### Outcomes

Seven patients (9%) had a history of temporary clozapine discontinuation for reasons including intolerable adverse effects, lack of efficacy and neutropenia. However, they had been restarted and were on clozapine therapy at the time of data collection. Rates of permanent discontinuation of clozapine therapy in the total patient population over the years were difficult to ascertain; however, our unpublished data have been consistent with the reported incidence of agranulocytosis (0.7-1%). 

Sedation and hypersalivation were the most commonly reported adverse events with an incidence of 34 % and 26%, respectively. Weight gain was reported in 7% of patients. Seven (9%) participants did not report any adverse event; the rest reported one or more adverse events. 

Number of hospitalizations was assessed for each patient prior to clozapine initiation and after clozapine initiation. In the pre-clozapine period more than 50% of the patients had at least 2 hospitalizations, this proportion decreased dramatically to 13% after clozapine was initiated. More than 55% of patients had no hospitalizations during clozapine therapy. 

Residual positive (i.e., hallucinations and psychotic symptoms) and negative symptoms (i.e., withdrawal and depression) were observed in 20-30% of patients treated with clozapine, which prompted the use of combination therapy in these patients.

## Discussion

This retrospective chart review study provides a snapshot of a Canadian outpatient population on clozapine therapy. This sample of patients diagnosed with schizophrenia represents a stable and well assisted outpatient population served by diagnostic and therapeutic services provided within an urban hospital facility in a Canadian city of approximately 700,000 residents. 

It was observed that 60% of the population had been taking clozapine for 5 years or longer, and more than 85% had been taking clozapine for 2 years or longer. This is consistent with previous data confirming that maintenance rates for clozapine are higher than those for SGAs (i.e., risperidone, olanzapine, quetiapine) [5,6]. This may be due to the constant contact provided through the required hematological testing.

Our data demonstrate a relatively high rate of clozapine monotherapy though lower than that shown in other studies. Notably, one study demonstrated clozapine monotherapy rates as highs as 89% [13].

Patients in our study were diagnosed with treatment-resistant schizophrenia and were appropriately prescribed clozapine; however, only 14 patients (21%) had tried 2 antipsychotic medications prior to clozapine with the majority (68%) having tried 3 or more agents. Also the subset of patients initiated on clozapine after 2005, showed similar profiles in terms of previous antipsychotic trials, only a trend toward shorter duration of other therapies before clozapine initiation was observed; however, due to the limited sample size no statistical analysis could be conducted. 

These findings are indicative of the hesitancy to switch patients to clozapine and its status as a last-resort treatment option. Several other studies have also demonstrated delays in clozapine initiation after diagnosis [12-14]. Reasons for such delays are often difficult to determine; however, concerns regarding the potentially life-threatening toxicity of clozapine probably play a major role in clinicians’ reluctance to prescribe this drug to their patients. Another factor can be patients’ reluctance in accepting a therapy that requires frequent blood testing. Furthermore, side effects such as weight gain, hypersalivation and sedation might be considered significant for some patients and can contribute also to a delay in clozapine prescribing. 

Adverse events were reported as recorded in the charts, no rating scales had been used and no systematic assessment was possible. Similarly, assessment of residual symptoms was extracted from the charts based on the therapist/psychiatrist notes. Since data were collected retrospectively, no consistent assessment of treatment effectiveness was possible. Nevertheless, it can be inferred that patients on clozapine monotherapy were well managed and “functional”. The proportion of individuals who needed clozapine augmentation therapy was consistent with reports in the literature where up to 50% of patients affected by treatment-resistant schizophrenia were not managed by clozapine alone and required combination therapy [15]. 

Our results are limited by the accuracy and completeness of the information contained in the medical charts and by the small sample size. However, the observational approach used provides real-world information regarding the use of clozapine in a Canadian outpatient population.

## Conclusion

Until 2010, the use of clozapine in patients with a diagnosis of schizophrenia was often delayed and more than 2 antipsychotic medications were tried for relatively long periods of time before patients were switched to clozapine, despite published evidence and clinical practice guidelines supporting the effectiveness of clozapine. Patients were often subjected to lengthy periods of ineffective therapy. It is desirable that adherence to new evidence-based guidelines regarding the pharmacological treatment of schizophrenia [16] will result in better patient outcomes in the future. 
